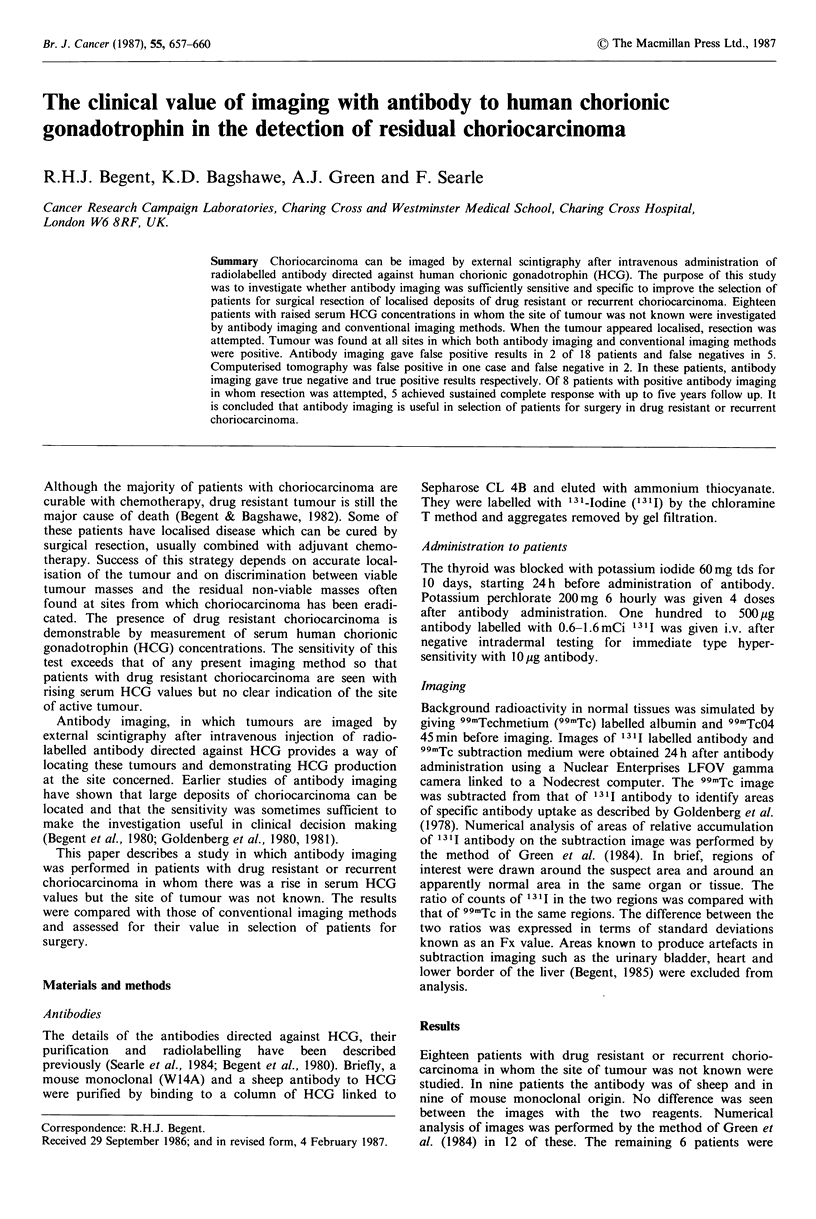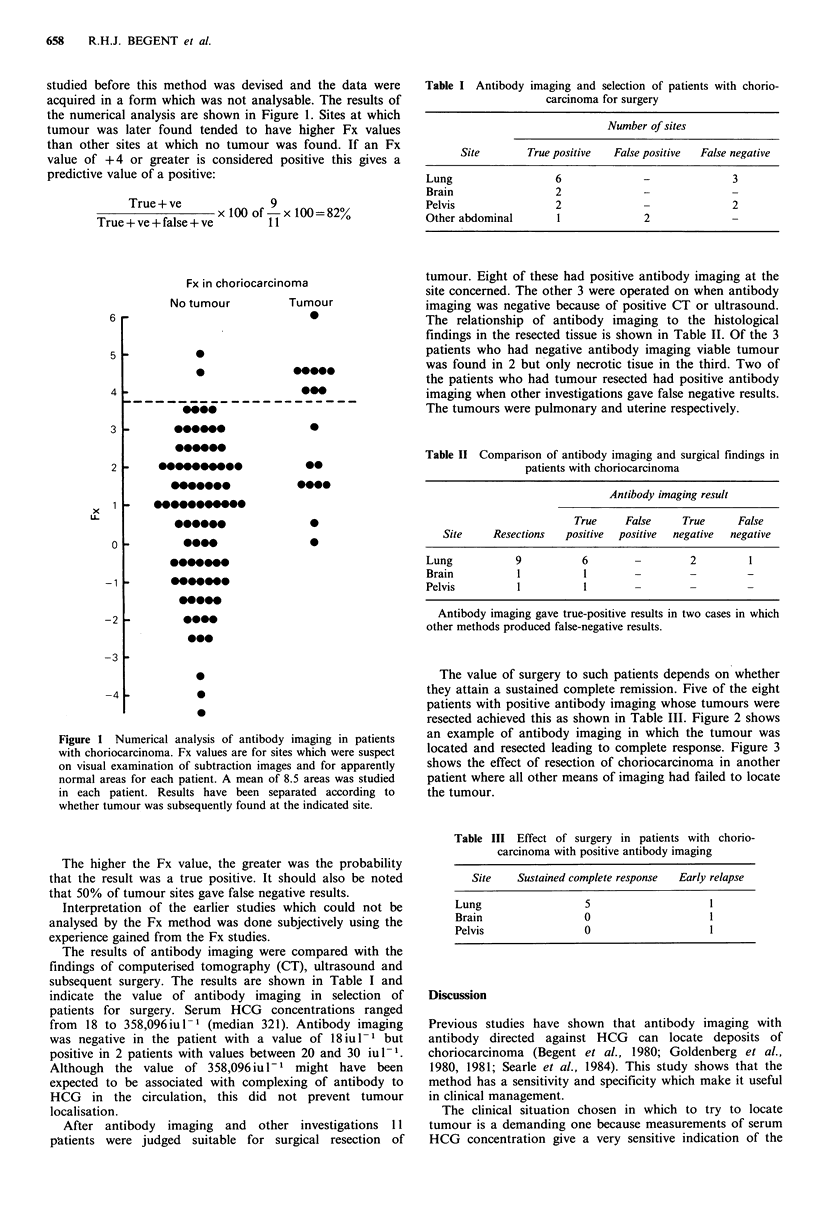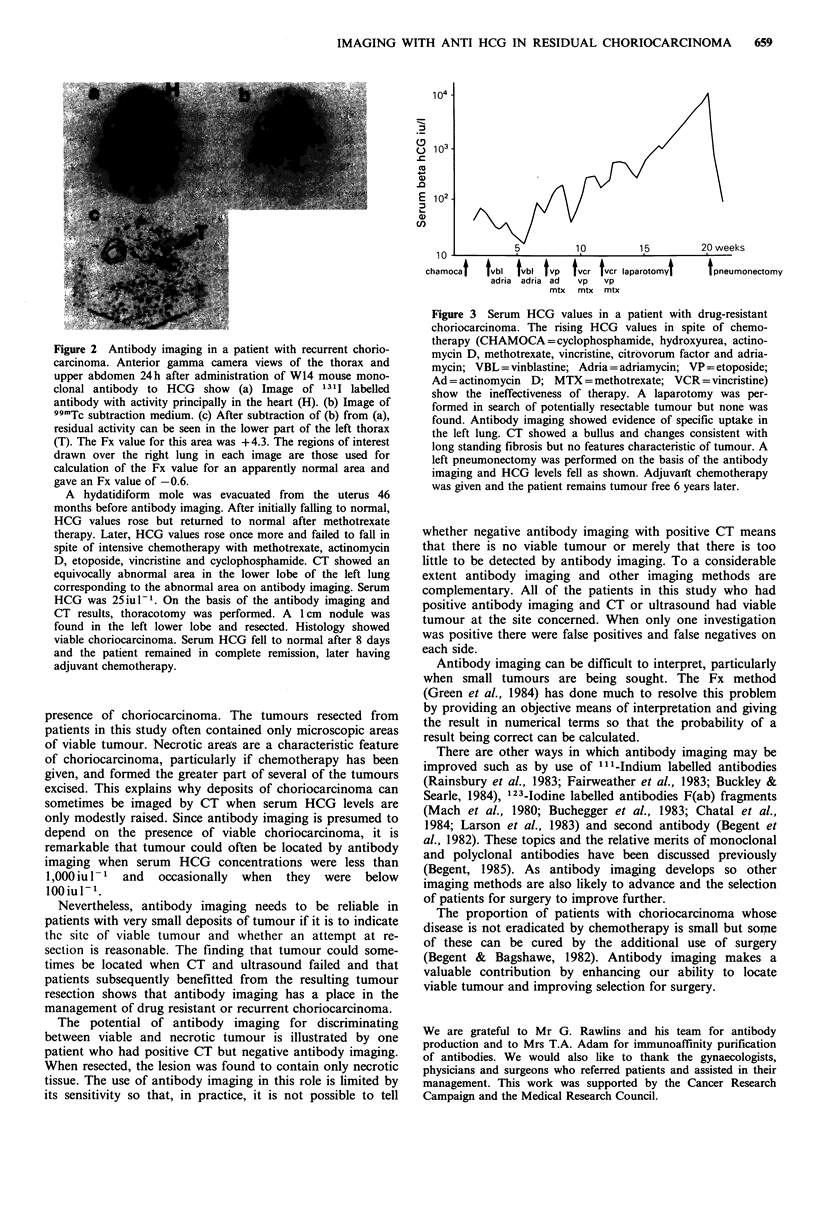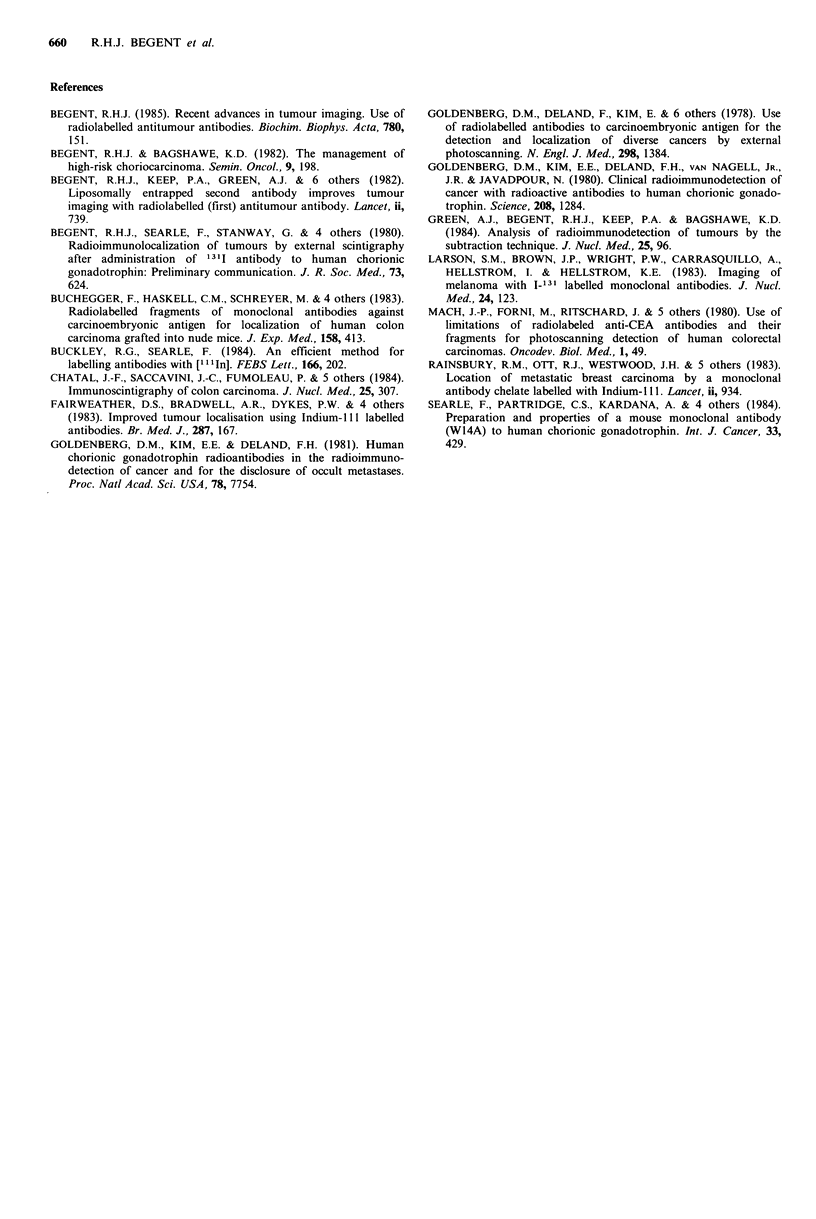# The clinical value of imaging with antibody to human chorionic gonadotrophin in the detection of residual choriocarcinoma.

**DOI:** 10.1038/bjc.1987.134

**Published:** 1987-06

**Authors:** R. H. Begent, K. D. Bagshawe, A. J. Green, F. Searle

## Abstract

**Images:**


*Br. J. Cancer* (1987), **55**, 657–660 © The Macmillan Press Ltd., 1987

### The clinical value of imaging with antibody to human chorionic gonadotrophin in the detection of residual choriocarcinoma

R.H.J. Begent, K.D. Bagshawe, A.J. Green and F. Searle

*Cancer Research Campaign Laboratories, Charing Cross and Westminster Medical School, Charing Cross Hospital, London W6 8RF, UK.*

**Summary** Choriocarcinoma can be imaged by external scintigraphy after intravenous administration of radiolabelled antibody directed against human chorionic gonadotrophin (HCG). The purpose of this study was to investigate whether antibody imaging was sufficiently sensitive and specific to improve the selection of patients for surgical resection of localised deposits of drug resistant or recurrent choriocarcinoma. Eighteen patients with raised serum HCG concentrations in whom the site of tumour was not known were investigated by antibody imaging and conventional imaging methods. When the tumour appeared localised, resection was attempted. Tumour was found at all sites in which both antibody imaging and conventional imaging methods were positive. Antibody imaging gave false positive results in 2 of 18 patients and false negatives in 5. Computerised tomography was false positive in one case and false negative in 2. In these patients, antibody imaging gave true negative and true positive results respectively. Of 8 patients with positive antibody imaging in whom resection was attempted, 5 achieved sustained complete response with up to five years follow up. It is concluded that antibody imaging is useful in selection of patients for surgery in drug resistant or recurrent choriocarcinoma.

Although the majority of patients with choriocarcinoma are curable with chemotherapy, drug resistant tumour is still the major cause of death (Begent & Bagshawe, 1982). Some of these patients have localised disease which can be cured by surgical resection, usually combined with adjuvant chemotherapy. Success of this strategy depends on accurate localisation of the tumour and on discrimination between viable tumour masses and the residual non-viable masses often found at sites from which choriocarcinoma has been eradicated. The presence of drug resistant choriocarcinoma is demonstrable by measurement of serum human chorionic gonadotrophin (HCG) concentrations. The sensitivity of this test exceeds that of any present imaging method so that patients with drug resistant choriocarcinoma are seen with rising serum HCG values but no clear indication of the site of active tumour.

Antibody imaging, in which tumours are imaged by external scintigraphy after intravenous injection of radiolabelled antibody directed against HCG provides a way of locating these tumours and demonstrating HCG production at the site concerned. Earlier studies of antibody imaging have shown that large deposits of choriocarcinoma can be located and that the sensitivity was sometimes sufficient to make the investigation useful in clinical decision making (Begent *et al.*, 1980; Goldenberg *et al.*, 1980, 1981).

This paper describes a study in which antibody imaging was performed in patients with drug resistant or recurrent choriocarcinoma in whom there was a rise in serum HCG values but the site of tumour was not known. The results were compared with those of conventional imaging methods and assessed for their value in selection of patients for surgery.

### Materials and methods

### *Antibodies*

The details of the antibodies directed against HCG, their purification and radiolabelling have been described previously (Searle *et al.*, 1984; Begent *et al.*, 1980). Briefly, a mouse monoclonal (W14A) and a sheep antibody to HCG were purified by binding to a column of HCG linked to Sepharose CL 4B and eluted with ammonium thiocyanate. They were labelled with $^{131}$-Iodine ($^{131}$I) by the chloramine T method and aggregates removed by gel filtration.

### *Administration to patients*

The thyroid was blocked with potassium iodide 60 mg tds for 10 days, starting 24 h before administration of antibody. Potassium perchlorate 200 mg 6 hourly was given 4 doses after antibody administration. One hundred to 500 $\mu$g antibody labelled with 0.6–1.6 mCi $^{131}$I was given i.v. after negative intradermal testing for immediate type hypersensitivity with 10 $\mu$g antibody.

### *Imaging*

Background radioactivity in normal tissues was simulated by giving $^{99m}$Techmetium ($^{99m}$Tc) labelled albumin and $^{99m}$Tc04 45 min before imaging. Images of $^{131}$I labelled antibody and $^{99m}$Tc subtraction medium were obtained 24 h after antibody administration using a Nuclear Enterprises LFOV gamma camera linked to a Nodecrest computer. The $^{99m}$Tc image was subtracted from that of $^{131}$I antibody to identify areas of specific antibody uptake as described by Goldenberg *et al.* (1978). Numerical analysis of areas of relative accumulation of $^{131}$I antibody on the subtraction image was performed by the method of Green *et al.* (1984). In brief, regions of interest were drawn around the suspect area and around an apparently normal area in the same organ or tissue. The ratio of counts of $^{131}$I in the two regions was compared with that of $^{99m}$Tc in the same regions. The difference between the two ratios was expressed in terms of standard deviations known as an Fx value. Areas known to produce artefacts in subtraction imaging such as the urinary bladder, heart and lower border of the liver (Begent, 1985) were excluded from analysis.

### Results

Eighteen patients with drug resistant or recurrent choriocarcinoma in whom the site of tumour was not known were studied. In nine patients the antibody was of sheep and in nine of mouse monoclonal origin. No difference was seen between the images with the two reagents. Numerical analysis of images was performed by the method of Green *et al.* (1984) in 12 of these. The remaining 6 patients were

Correspondence: R.H.J. Begent.
Received 29 September 1986; and in revised form, 4 February 1987.

studied before this method was devised and the data were acquired in a form which was not analysable. The results of the numerical analysis are shown in Figure 1. Sites at which tumour was later found tended to have higher Fx values than other sites at which no tumour was found. If an Fx value of +4 or greater is considered positive this gives a predictive value of a positive:

$$\frac{\text{True}+\text{ve}}{\text{True}+\text{ve}+\text{false}+\text{ve}}\times 100 \text{ of } \frac{9}{11}\times 100=82\%$$

**Figure 1** Numerical analysis of antibody imaging in patients with choriocarcinoma. Fx values are for sites which were suspect on visual examination of subtraction images and for apparently normal areas for each patient. A mean of 8.5 areas was studied in each patient. Results have been separated according to whether tumour was subsequently found at the indicated site.

The higher the Fx value, the greater was the probability that the result was a true positive. It should also be noted that 50% of tumour sites gave false negative results.

Interpretation of the earlier studies which could not be analysed by the Fx method was done subjectively using the experience gained from the Fx studies.

The results of antibody imaging were compared with the findings of computerised tomography (CT), ultrasound and subsequent surgery. The results are shown in Table I and indicate the value of antibody imaging in selection of patients for surgery. Serum HCG concentrations ranged from 18 to 358,096 iu $l^{-1}$ (median 321). Antibody imaging was negative in the patient with a value of 18 iu $l^{-1}$ but positive in 2 patients with values between 20 and 30 iu $l^{-1}$. Although the value of 358,096 iu $l^{-1}$ might have been expected to be associated with complexing of antibody to HCG in the circulation, this did not prevent tumour localisation.

After antibody imaging and other investigations 11 patients were judged suitable for surgical resection of tumour. Eight of these had positive antibody imaging at the site concerned. The other 3 were operated on when antibody imaging was negative because of positive CT or ultrasound. The relationship of antibody imaging to the histological findings in the resected tissue is shown in Table II. Of the 3 patients who had negative antibody imaging viable tumour was found in 2 but only necrotic tisue in the third. Two of the patients who had tumour resected had positive antibody imaging when other investigations gave false negative results. The tumours were pulmonary and uterine respectively.

**Table I** Antibody imaging and selection of patients with choriocarcinoma for surgery

| | *Number of sites* | | |
|---|---|---|---|
| *Site* | *True positive* | *False positive* | *False negative* |
| Lung | 6 | – | 3 |
| Brain | 2 | – | – |
| Pelvis | 2 | – | 2 |
| Other abdominal | 1 | 2 | – |

**Table II** Comparison of antibody imaging and surgical findings in patients with choriocarcinoma

| | | *Antibody imaging result* | | | |
|---|---|---|---|---|---|
| *Site* | *Resections* | *True positive* | *False positive* | *True negative* | *False negative* |
| Lung | 9 | 6 | – | 2 | 1 |
| Brain | 1 | 1 | – | – | – |
| Pelvis | 1 | 1 | – | – | – |

Antibody imaging gave true-positive results in two cases in which other methods produced false-negative results.

The value of surgery to such patients depends on whether they attain a sustained complete remission. Five of the eight patients with positive antibody imaging whose tumours were resected achieved this as shown in Table III. Figure 2 shows an example of antibody imaging in which the tumour was located and resected leading to complete response. Figure 3 shows the effect of resection of choriocarcinoma in another patient where all other means of imaging had failed to locate the tumour.

**Table III** Effect of surgery in patients with choriocarcinoma with positive antibody imaging

| *Site* | *Sustained complete response* | *Early relapse* |
|---|---|---|
| Lung | 5 | 1 |
| Brain | 0 | 1 |
| Pelvis | 0 | 1 |

### Discussion

Previous studies have shown that antibody imaging with antibody directed against HCG can locate deposits of choriocarcinoma (Begent *et al.*, 1980; Goldenberg *et al.*, 1980, 1981; Searle *et al.*, 1984). This study shows that the method has a sensitivity and specificity which make it useful in clinical management.

The clinical situation chosen in which to try to locate tumour is a demanding one because measurements of serum HCG concentration give a very sensitive indication of the

**Figure 2** Antibody imaging in a patient with recurrent choriocarcinoma. Anterior gamma camera views of the thorax and upper abdomen 24 h after administration of W14 mouse monoclonal antibody to HCG show (a) Image of $^{131}I$ labelled antibody with activity principally in the heart (H). (b) Image of $^{99m}Tc$ subtraction medium. (c) After subtraction of (b) from (a), residual activity can be seen in the lower part of the left thorax (T). The Fx value for this area was +4.3. The regions of interest drawn over the right lung in each image are those used for calculation of the Fx value for an apparently normal area and gave an Fx value of −0.6.

A hydatidiform mole was evacuated from the uterus 46 months before antibody imaging. After initially falling to normal, HCG values rose but returned to normal after methotrexate therapy. Later, HCG values rose once more and failed to fall in spite of intensive chemotherapy with methotrexate, actinomycin D, etoposide, vincristine and cyclophosphamide. CT showed an equivocally abnormal area in the lower lobe of the left lung corresponding to the abnormal area on antibody imaging. Serum HCG was 25 iu l$^{-1}$. On the basis of the antibody imaging and CT results, thoracotomy was performed. A 1 cm nodule was found in the left lower lobe and resected. Histology showed viable choriocarcinoma. Serum HCG fell to normal after 8 days and the patient remained in complete remission, later having adjuvant chemotherapy.

presence of choriocarcinoma. The tumours resected from patients in this study often contained only microscopic areas of viable tumour. Necrotic areas are a characteristic feature of choriocarcinoma, particularly if chemotherapy has been given, and formed the greater part of several of the tumours excised. This explains why deposits of choriocarcinoma can sometimes be imaged by CT when serum HCG levels are only modestly raised. Since antibody imaging is presumed to depend on the presence of viable choriocarcinoma, it is remarkable that tumour could often be located by antibody imaging when serum HCG concentrations were less than 1,000 iu l$^{-1}$ and occasionally when they were below 100 iu l$^{-1}$.

Nevertheless, antibody imaging needs to be reliable in patients with very small deposits of tumour if it is to indicate the site of viable tumour and whether an attempt at resection is reasonable. The finding that tumour could sometimes be located when CT and ultrasound failed and that patients subsequently benefitted from the resulting tumour resection shows that antibody imaging has a place in the management of drug resistant or recurrent choriocarcinoma.

The potential of antibody imaging for discriminating between viable and necrotic tumour is illustrated by one patient who had positive CT but negative antibody imaging. When resected, the lesion was found to contain only necrotic tissue. The use of antibody imaging in this role is limited by its sensitivity so that, in practice, it is not possible to tell

**Figure 3** Serum HCG values in a patient with drug-resistant choriocarcinoma. The rising HCG values in spite of chemotherapy (CHAMOCA = cyclophosphamide, hydroxyurea, actinomycin D, methotrexate, vincristine, citrovorum factor and adriamycin; VBL = vinblastine; Adria = adriamycin; VP = etoposide; Ad = actinomycin D; MTX = methotrexate; VCR = vincristine) show the ineffectiveness of therapy. A laparotomy was performed in search of potentially resectable tumour but none was found. Antibody imaging showed evidence of specific uptake in the left lung. CT showed a bullus and changes consistent with long standing fibrosis but no features characteristic of tumour. A left pneumonectomy was performed on the basis of the antibody imaging and HCG levels fell as shown. Adjuvant chemotherapy was given and the patient remains tumour free 6 years later.

whether negative antibody imaging with positive CT means that there is no viable tumour or merely that there is too little to be detected by antibody imaging. To a considerable extent antibody imaging and other imaging methods are complementary. All of the patients in this study who had positive antibody imaging and CT or ultrasound had viable tumour at the site concerned. When only one investigation was positive there were false positives and false negatives on each side.

Antibody imaging can be difficult to interpret, particularly when small tumours are being sought. The Fx method (Green *et al.*, 1984) has done much to resolve this problem by providing an objective means of interpretation and giving the result in numerical terms so that the probability of a result being correct can be calculated.

There are other ways in which antibody imaging may be improved such as by use of $^{111}$-Indium labelled antibodies (Rainsbury *et al.*, 1983; Fairweather *et al.*, 1983; Buckley & Searle, 1984), $^{123}$-Iodine labelled antibodies F(ab) fragments (Mach *et al.*, 1980; Buchegger *et al.*, 1983; Chatal *et al.*, 1984; Larson *et al.*, 1983) and second antibody (Begent *et al.*, 1982). These topics and the relative merits of monoclonal and polyclonal antibodies have been discussed previously (Begent, 1985). As antibody imaging develops so other imaging methods are also likely to advance and the selection of patients for surgery to improve further.

The proportion of patients with choriocarcinoma whose disease is not eradicated by chemotherapy is small but some of these can be cured by the additional use of surgery (Begent & Bagshawe, 1982). Antibody imaging makes a valuable contribution by enhancing our ability to locate viable tumour and improving selection for surgery.

We are grateful to Mr G. Rawlins and his team for antibody production and to Mrs T.A. Adam for immunoaffinity purification of antibodies. We would also like to thank the gynaecologists, physicians and surgeons who referred patients and assisted in their management. This work was supported by the Cancer Research Campaign and the Medical Research Council.

### References

BEGENT, R.H.J. (1985). Recent advances in tumour imaging. Use of radiolabelled antitumour antibodies. *Biochim. Biophys. Acta*, **780**, 151.

BEGENT, R.H.J. & BAGSHAWE, K.D. (1982). The management of high-risk choriocarcinoma. *Semin. Oncol.*, **9**, 198.

BEGENT, R.H.J., KEEP, P.A., GREEN, A.J. & 6 others (1982). Liposomally entrapped second antibody improves tumour imaging with radiolabelled (first) antitumour antibody. *Lancet*, **ii**, 739.

BEGENT, R.H.J., SEARLE, F., STANWAY, G. & 4 others (1980). Radioimmunolocalization of tumours by external scintigraphy after administration of $^{131}$I antibody to human chorionic gonadotrophin: Preliminary communication. *J. R. Soc. Med.*, **73**, 624.

BUCHEGGER, F., HASKELL, C.M., SCHREYER, M. & 4 others (1983). Radiolabelled fragments of monoclonal antibodies against carcinoembryonic antigen for localization of human colon carcinoma grafted into nude mice. *J. Exp. Med.*, **158**, 413.

BUCKLEY, R.G., SEARLE, F. (1984). An efficient method for labelling antibodies with [$^{111}$In]. *FEBS Lett.*, **166**, 202.

CHATAL, J.-F., SACCAVINI, J.-C., FUMOLEAU, P. & 5 others (1984). Immunoscintigraphy of colon carcinoma. *J. Nucl. Med.*, **25**, 307.

FAIRWEATHER, D.S., BRADWELL, A.R., DYKES, P.W. & 4 others (1983). Improved tumour localisation using Indium-111 labelled antibodies. *Br. Med. J.*, **287**, 167.

GOLDENBERG, D.M., KIM, E.E. & DELAND, F.H. (1981). Human chorionic gonadotrophin radioantibodies in the radioimmunodetection of cancer and for the disclosure of occult metastases. *Proc. Natl Acad. Sci. USA*, **78**, 7754.

GOLDENBERG, D.M., DELAND, F., KIM, E. & 6 others (1978). Use of radiolabelled antibodies to carcinoembryonic antigen for the detection and localization of diverse cancers by external photoscanning. *N. Engl. J. Med.*, **298**, 1384.

GOLDENBERG, D.M., KIM, E.E., DELAND, F.H., VAN NAGELL, JR., J.R. & JAVADPOUR, N. (1980). Clinical radioimmunodetection of cancer with radioactive antibodies to human chorionic gonadotrophin. *Science*, **208**, 1284.

GREEN, A.J., BEGENT, R.H.J., KEEP, P.A. & BAGSHAWE, K.D. (1984). Analysis of radioimmunodetection of tumours by the subtraction technique. *J. Nucl. Med.*, **25**, 96.

LARSON, S.M., BROWN, J.P., WRIGHT, P.W., CARRASQUILLO, A., HELLSTROM, I. & HELLSTROM, K.E. (1983). Imaging of melanoma with I-$^{131}$ labelled monoclonal antibodies. *J. Nucl. Med.*, **24**, 123.

MACH, J.-P., FORNI, M., RITSCHARD, J. & 5 others (1980). Use of limitations of radiolabeled anti-CEA antibodies and their fragments for photoscanning detection of human colorectal carcinomas. *Oncodev. Biol. Med.*, **1**, 49.

RAINSBURY, R.M., OTT, R.J., WESTWOOD, J.H. & 5 others (1983). Location of metastatic breast carcinoma by a monoclonal antibody chelate labelled with Indium-111. *Lancet*, **ii**, 934.

SEARLE, F., PARTRIDGE, C.S., KARDANA, A. & 4 others (1984). Preparation and properties of a mouse monoclonal antibody (W14A) to human chorionic gonadotrophin. *Int. J. Cancer*, **33**, 429.